# Development of the HPLC Method for Simultaneous Determination of Lidocaine Hydrochloride and Tribenoside Along with Their Impurities Supported by the QSRR Approach

**DOI:** 10.1007/s10337-012-2339-9

**Published:** 2012-11-01

**Authors:** Alina Plenis, Lucyna Konieczna, Natalia Miękus, Tomasz Bączek

**Affiliations:** Department of Pharmaceutical Chemistry, Medical University of Gdańsk, Hallera 107, 80-416 Gdańsk, Poland

**Keywords:** Column Liquid chromatography, Column classification, Quantitative structure-retention relationships, Factor analysis, Lidocaine hydrochloride, Tribenoside, Impurities

## Abstract

A new liquid chromatographic (LC) method for simultaneous determination of lidocaine hydrochloride (LH) and tribenoside (TR) along with their related compounds in pharmaceutical preparations is described. Satisfactory LC separation of all analytes after the liquid–liquid extraction (LLE) procedure with ethanol was performed on a C_18_ column using a gradient elution of a mixture of acetonitrile and 0.1 % orthophosphoric acid as the mobile phase. The procedure was validated according to the ICH guidelines. The limits of detection (LOD) and quantification (LOQ) were 4.36 and 13.21 μg mL^−1^ for LH, 7.60 and 23.04 μg mL^−1^ for TR, and below 0.11 and 0.33 μg mL^−1^ for their impurities, respectively. Intra- and inter-day precision was below 1.97 %, whereas accuracy for all analytes ranged from 98.17 to 101.94 %. The proposed method was sensitive, robust, and specific allowing reliable simultaneous quantification of all mentioned compounds. Moreover, a comparative study of the RP-LC column classification based on the quantitative structure-retention relationships (QSRR) and column selectivity obtained in real pharmaceutical analysis was innovatively applied using factor analysis (FA). In the column performance test, the analysis of LH and TR in the presence of their impurities was carried out according to the developed method with the use of 12 RP-LC stationary phases previously tested under the QSRR conditions. The obtained results confirmed that the classes of the stationary phases selected in accordance with the QSRR models provided comparable separation for LH, TR, and their impurities. Hence, it was concluded that the proposed QSRR approach could be considered a supportive tool in the selection of the suitable column for the pharmaceutical analysis.

## Introduction

Tribenoside (TR), chemically a mixture of the α- and β-anomers of ethyl 3,5,6-tri-*O*-benzyl-d-glucofuranoside (TRα, TRβ), demonstrates anti-inflammatory, mild analgesic, antitoxic, wound-healing, fibrinolysis-promoting, anti-arthrotic, amine-release-inhibitory, membrane-stabilising, and venotropic properties. The mechanism of pharmacological activity of lidocaine hydrochloride (LH), chemically 2-(diethylamino)-*N*-(2,6-dimethylphenyl)acetamide hydrochloride, is different. This drug belongs to the widest used local anaesthetic agents applied in regional management of major pain, administered spinally and epidurally or peripherally. Both substances are often combined in pharmaceutical preparations to treat haemorrhoids because the lidocaine component helps to provide rapid symptomatic relief.

Both TR and LH are official drugs in the European Pharmacopeia (Ph. Eur.) [1] and British Pharmacopeia (BP) [2]. Both monographs describe the LC procedures for the determination of LH and its two impurities: 2,6-dimethylaniline (DMA) and 2-chloro-2′,6′-acetoxylidide (CAX), as well as TR along with its related compounds: benzaldehyde (BA), dibenzyl ether (DBE), plus tribenoside impurity A (TRA). Unfortunately, these methods are based on different chromatographic conditions making simultaneous determination of LH and TR in a single LC run time impossible. Although many HPLC procedures for the analysis of LH in pharmaceutical preparations have been published in literature [3–11], only few papers have reported methods for quantification of the substance along with its impurities [1, 2, 4, 5, 9–11]. As concerns TR, publications concern only the HPLC [12], gas–liquid chromatography (GLC) [13], and spectrophotometry [14] and exclude any related compounds. Thus, to the best of our knowledge no method has so far been reported for simultaneous quantitative determination of LH and TR along with their impurities in pharmaceutical products.

It is also common knowledge that analysts often face the problem of appropriate column selection when the stationary phase used for the development of the method is not available in the laboratory or anywhere in the market. Furthermore, the properties of the required stationary phase can also change over the storage time or upon usage. All those factors may cause that the columns will not yield satisfactory results. To overcome the problem many general test methods have been extensively investigated to characterise the RP-LC columns over the last two decades [15–28]. Among them, the QSRR based on two mathematical models seems to be an interesting approach [21–28]. The first QSRR model relates log *k*_w_ as the retention factor *k* of the analyte extrapolated to the virtual mobile phase of pure water or pure buffer, to the theoretically calculated logarithms of *n*-octanol/water partition coefficients (*c*log *P*) [23].

1 $$ \log \,k_{\text{w}} = a_{1} + a_{2} c\log P $$

where *a*_1_ and *a*_4_ are the regression coefficients

The second model relates log *k*_w_ of the analyte to three calculated structural descriptors of the molecular modelling such as the total dipole moment *μ*, the electron excess charge of the most negatively charged atom *δ*_Min_, and the water-accessible molecular surface area *A*_WAS._

2 $$ \log \,k_{\text{w}} = a^{\prime}_{1} + a^{\prime}_{2} \mu + a^{\prime}_{3} \delta_{\text{Min}} + a^{\prime}_{4} A_{\text{WAS}} $$

where *a*_1_^′^ to *a*_4_^′^ are the regression coefficients. Unfortunately, only one paper has focused on the correlation of the theoretical QSRR results and the column performance in real biomedical analyse [28]. Thus, there is the need to conduct comparative studies to investigate whether the use of the QSRR increases the probability of appropriate column selection for the specific pharmaceutical separation.

Therefore, the aim of the study was to develop a simple, sensitive and accurate HPLC method for simultaneous determination of LH and TR along with their related compounds in pharmaceutical preparations. Another objective was to examine the reliability of the QSRR results in facilitating column selection against column performance in real pharmaceutical analysis. As a case study, LH and TR and their impurities were quantified in accordance with the developed method on 12 stationary phases previously tested under the QSRR conditions. Next, the retention parameters of the analysed substances such as the retention time (*t*_R_), and the resolution of the peaks of interest (*R*_s_) were calculated for all stationary phases providing a detailed description of the column performance in real analysis. Moreover, the values of the Ph. Eur. system suitability test (SST) required for LH and TR were identified for all tested columns. Then, the systematic information obtained from the set of the QSRR regression parameters used in characteristics of the stationary phases was correlated with the column performance in real pharmaceutical separation in a factor analysis (FA). The assay was conducted to check whether the column classes closely related in terms of their QSRR characteristics demonstrated similar separation for LH and TR.

## Experimental

### Reagents

The test analytes used to derive the QSRR model for the tested stationary phases were as follows: anisole (99 %), benzamide (98 %), benzonitrile (99.9 %), benzyl chloride (99 %), 4-cyanophenol (98 %), indole (99 %), 1-naphthylacetonitrile (97 %), phenanthrene (97 %), and pyrene (99 %), all obtained from Sigma-Aldrich (St. Louis, MO, USA); biphenyl (99 %), 2,2^′^-dinaphthyl ether (98 %), indazole (98 %), naphthalene (99.5 %), and 2-naphthol (99 %) supplied by Lancaster (Newgate, UK); and benzene (99.5 %) purchased from POCH (Gliwice, Poland). The degree of purity of the used chemicals is given in brackets. The analytes applied to develop the LC assay of LH and TR along with their impurities, to name e.g. DMA (99 %), CAX (99 %), TR (98 %), and TRA (98 %), were obtained from Sigma-Aldrich (St. Louis, MO, USA). LH (97 %) was delivered by Moehs Catalana (Barcelona, Spain), while BA (98 %) and DBE (98 %) were supplied by Fluka Chemie AG (Buchs, Switzerland). Ethanol and 85 % orthophosphoric acid, both of the analytical grade, were delivered by POCH (Gliwice, Poland). Acetonitrile and methanol of the HPLC grade were purchased from Merck (Darmstadt, Germany). All solvents and reagents were used as received without further purification. Water was purified in the Milli-Q Water Purification System (Millipore Corporation, Bedford, MA, USA). The Procto-Glyvenol^®^ cream (Novartis, Pardubice, Czech Republic) and Procto-Hemolan cream (Aflofarm, Ksawerów, Poland), both labelled to contain 2 % of LH and 5 % of TR, i.e. nominally 20 mg of LH and 50 mg of TR in 1 g, were both received within their shelf-life period. The 12 tested RP-LC columns examined in this study (Table 1) were donated by the manufacturers or suppliers.

**Table 1:** List of the RP-LC columns tested in the study

No.	Manufacturer/supplier	Name of the column	Length (mm)	Internal diameter (mm)	Particle size (μm)	Abbreviations
1	Varian	Varian C18	150	4.6	5	VR-150
2	Imtakt	Unison US-C18	125	4.6	5	UN-125
3	Knauer	Nucleosil 100-5 C18	250	4.0	5	NU18-250/5
4	Knauer	Nucleosil 100-5 C18	125	4.0	10	NU18-125/10
5	Macherey–Nagel	Nucleosil 100-7 C8	250	4.0	5	NU8-250/5
6	Knauer	Nucleosil 100-5 C18	125	4.0	5	NU18-125/5
7	Supelco	Discovery HS-C18	150	4.6	5	DI-150
8.	Supelco	Discovery HS-C18	250	4.6	5	DI-250
9	GL Sciences	Inertsil ODS-3	150	4.6	5	IN-150
10	Merck	LiChrospher RP-18	250	4.0	5	LI-250
11	Imtakt	Cadenza CD-C18	125	4.6	3	CA-125
12	Advanced Chrom.Tech./Achrom	ACE C18	150	4.6	5	AC-150

### Chromatographic Conditions

The analyses were performed on the ACME 9000 system (Younglin Instrument Corporation, Anyang, the Republic of Korea) equipped with a pump (SP 930D), variable wavelength UV/VIS detector (UV 730D), autosampler, thermostat (CTS30), and the AutoChro-3000 Chromatography Data System for data acquisition.

#### LC Conditions for the QSRR Method

Chromatographic separation proceeded with the gradient elution of water (solvent A) and methanol (solvent B), at the concentration changing from 5 to 100 % B and the gradient times *t*_*G*_ of 10 and 30 min. Next, the following gradient program was used in both LC runs: (i) 100 % B for 5 min, (ii) 5 % B for 15 min to achieve column equilibration. The flow rate was 1 mL min^−1^. The column temperature was maintained at 40 °C, and UV detection was conducted at 254 nm. A sample volume of 20 μL was injected into the HPLC system.

#### LC Conditions for the Determination of LH and TR along with Their Impurities

The chromatographic analysis was carried out on the Varian C_18_ column (150 × 4.6 mm, 5 μm) (VR-150) with the gradient elution of 0.1 % orthophosphoric acid (solvent A) and acetonitrile (solvent B) under the following conditions: (i) 0–5 min, 90 % solvent A isocratic; (ii) 5–10 min, linear to 45 % A; (iii) 10–20 min, linear to 0 % A; (iv) 20–35 min, 90 % A isocratic for column equilibration. All chromatographic measurements were conducted at 45 °C. The flow rate was 1 mL min^−1^. LH and its related compounds (DMA, CAX) were UV-monitored at 230 nm while TR and its impurities (BA, DBE and TRA) at 254 nm. The injection volume was 30 μL.

### Standard Solutions

The LH stock solution (5 mg mL^−1^) was prepared in mixture of methanol and water (1:1, v/v), while the stock solutions of DMA and CAX, both at the concentration of 1 mg mL^−1^, were prepared in methanol. The stock solutions of TR (12.5 mg mL^−1^) and its impurities (BA, DBE and TRA—at the level of 1 mg mL^−1^ each) were prepared in methanol. All stock solutions were stored in the dark at 4 °C and protected from light. The calibration standard solutions of LH in the range of 100–300 μg mL^−1^ and its two impurities in the range of 0.2–6 μg mL^−1^ each, as well as the calibration standard solutions of TR in the range of 250–750 μg mL^−1^ and its three related compounds in the range of 0.5–15 μg mL^−1^ each, were prepared by diluting the stock solutions of the compounds of the interest as appropriate.

The calibration standard samples, quality control samples (QCs), and cream samples were analysed in the same manner. The concentration of each analyte was calculated from the appropriate linear regression equation.

### Sample Preparation

The accurately weighed 1 g of either the Procto-Glyvenol^®^cream, or Procto-Hemolan cream was transferred to a 25 mL volumetric flask, dissolved in about 24 mL of ethanol, and shaken mechanically for 15 min. Next, the volume in the flask was adjusted with ethanol and the resulting mixture transported to an ultrasonic bath where the dissolution process was continued for 15 min. Then, the sample was frozen for 1 h at −20 °C and the solution filtered using an 0.45 μm syringe filtration disk (Whatman 25 mm GD/X glass microfiber GMF) from Whatman, Middlesex, Great Britain. After centrifugation for 8 min (7,000*g*) the obtained mixture was transferred to a clean test tube and diluted five times with ethanol. Finally, the whole solution was centrifuged for 8 min (7,000*g*) and a 20 μL aliquot was injected into the LC system.

### Method Validation

**Fig. 1:**
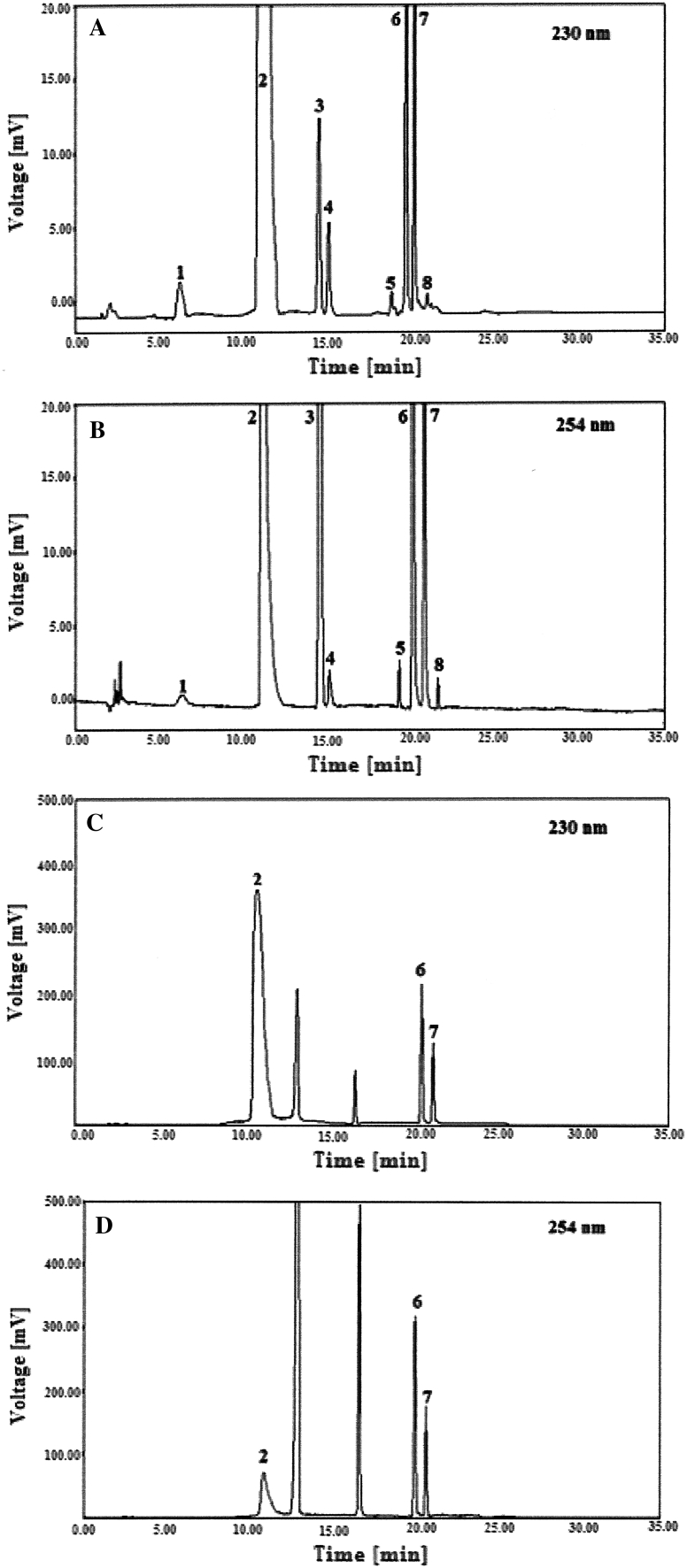
Representative chromatograms of the laboratory prepared mixtures at the concentrations of: 1.0 μg mL^−1^ for DMA (*1*), 200 μg mL^−1^ for LH (*2*); 2.5 μg mL^−1^ for BA (*3*); 1.0 μg mL^−1^ for CAX (*4*); 2.5 μg mL^−1^ for DBE (*5*); 500 μg mL^−1^ for TR [TRβ (*6*), TRα (*7*)] and 2.5 μg mL^−1^ for TRA (*8*), monitored at 230 nm (**a**) and 254 nm (**b**). Representative chromatograms of the Procto-Glyvenol^®^cream sample containing LH (200 μg mL^−1^) and TR (500 μg mL^−1^) monitored at 230 nm (**c**) and 254 nm (**d**), respectively

The validation of the optimized method was performed in accordance with the ICH guidelines Q2(R1) [29]. The obtained validation data are reported in Fig. 1 and Table 2, respectively.

**Table 2:** Summary of the validation data (*n* = 6) and performance data (*n* = 7) obtained by LC for simultaneous determination of LH, TR, and their impurities

Validation parameters	LH	DMA	CAX		TR	BA	DBE	TRA
Linearity (μg mL^−1^)	100–300	0.2–6			250–750	0.5–15		
Equation parameter								
Slope	27.603 ± 0.1872	7.704 ± 0.030		37.045 ± .176	1.5678 ± 0.0074	158.7 ± 0.61	2.5996 ± 0.0099	1.4005 ± 0.0428
Intercept	240.08 ± 36.46	1.2563 ± 0.1023		43.61±	62.022 ± 3.612	35.457 ± 5.213	1.5276 ± 0.0850	0.225 ± 0.005
Standard error	39.168	0.154		0.896	3.880	7.889	0.128	0.065
Correlation coefficient (*r*)	0.9998	0.9999		0.9998	0.9998	0.9997	0.9999	0.9998
LOD (μg mL^−1^)	4.36	0.04		0.05	7.60	0.11	0.11	0.10
LOQ (μg mL^−1^)	13.21	0.13		0.16	23.04	0.33	0.33	0.30
Precision RSD (%)								
Intra-day	0.28–1.78	0.40–1.97		0.29–1.69	0.34–1.86	0.29–1.80	0.27–1.93	0.28–1.88
Inter-day	0.35–1.84	0.38–1.96		0.25–1.83	0.36–1.89	0.25–1.82	0.33–1.96	0.36–1.94
Accuracy (%)								
Intra-day	98.58–101.45	98.26–101.58		99.04–100.91	99.08–101.69	99.14–100.94	98.17–101.85	99.02–101.66
Inter-day	99.10–101.12	98.39–101.92		98.87–101.34	98.55–101.76	99.03–101.12	98.25–100.44	98.59–101.94
Drugs in dosage forms								
Procto-Glyvenol^®^cream^a^								
Recovery^a^ [%]	100.21 ± 0.84	n.d.		n.d.	99.93 ± 0.71	n.d.	n.d.	n.d.
RSD %	0.84				0.71			
Procto-Hemolan cream^a^								
Recovery^a^ [%]	99.85 ± 0.89				100.52 ± 0.81			
RSD %	0.89				0.81			

^a^The percent of found to declared composition for seven replicate determination (mean ± SD); *n.d*. not detected (<LOD)

### Column Examination

#### Column Classification by QSRR

The calculation of the experimental log *k*_w_ for 15 test analytes used in the QSRR models was conducted on the DryLab computer simulation software (Molnar-Institute, Berlin, Germany). In the calculations, the *t*_R_ of the test analytes determined in two gradient LC runs, and the *t*_R_ of uracil were used, respectively to evaluate the column dead time. The logarithms of *c*log *P* used were established by employing the ALOGPS 2.1 program available online at http://www.vcclab.org. Other structural descriptors of the molecular modelling such as *μ*, *δ*_Min_, and *A*_WAS_ were calculated in the HyperChem program with the extension of ChemPlus (HyperCube, Waterloo, Canada) [23]. The multiple linear regression equations for 12 stationary phases were derived from Eqs. 1 and 2 on the Microsoft Excel software (Microsoft, Redmond, WA, USA). The regression coefficients (±standard deviations), multiple correlation coefficients (*r*), standard errors of the equation estimates (*s*), significance levels of each term and the whole equation (*p*), and the values obtained in the Fisher’s *F*-test of significance (Fisher’s *F*-test) are presented in Tables 3 and 4, respectively.

**Table 3:** Coefficients *a*_1_ and *a*_2_ (±standard deviations) with their significance levels, *p* (underneath in parenthesis), and statistical parameters, *r*, *s*, Fisher’s *F*-test and *p* (see text for explanation), of the regression equations of the forms: $\log \,k_{\text{w}} = a_{1} + a_{2} c\log P$, provided by the QSRR for twelve tested columns

No.	Analytical column	*a*_1_	*a*_2_	*r*	*s*	Fisher’s* F*-test	*p*
1	VA-150	0.445 (±0.112)	0.722 (±0.036) (*p* = 4E−11)	0.9842	0.190	401.0	4E−11
2	NU18-125/5	0.029 (±0.089)	0.949 (±0.028) (*p* = 6E−14)	0.9942	0.150	1105.9	6E−14
3	NU18-250/5	−0.719 (±0.091)	1.046 (±0.029) (*p* = 2E−14)	0.9949	0.155	1267.3	2E−14
4	NU8-250/5	0.002 (±0.105)	0.786 (±0.034) (*p* = 5E−12)	0.9883	0.177	546.8	5E−12
5	NU18-125/10	0.184 (±0.060)	0.912 (±0.019) (*p* = 6E−16)	0.9971	0.101	2247.5	6E−16
6	UN-125	0.670 (±0.118)	0.622 (±0.038) (*p* = 5E−10)	0.9765	0.201	266.9	5E−10
7	IN-150	0.640 (±0.096)	0.720 (±0.031) (*p* = 5E−12)	0.9883	0.163	544.1	5E−12
8	AC-150	0.746 (±0.084)	0.550 (±0.027) (*p* = 3E−11)	0.9847	0.142	415.2	3E−11
9	DI-150	0.514 (±0.095)	0.750 (±0.030) (*p* = 3E−12)	0.9895	0.160	607.2	3E−12
10	DI-250	0.687 (±0.108)	0.675 (±0.035) (*p* = 6E−11)	0.9831	0.183	376.2	6E−11
11	CA-125	0.795 (±0.143)	0.668 (±0.046) (*p* = 2 E-09)	0.9707	0.242	212.3	2E−09
12	LI-250	0.678 (±0.078)	0.588 (±0.025) (*p* = 5E−12)	0.9883	0.132	548.2	5E−12

**Table 4:** Coefficients *a*_1_^′^–*a*_4_^′^ (±standard deviations) with their significance levels, *p* (underneath in parenthesis), and statistical parameters, *r*, *s*, Fisher’s *F*-test and *p* (see text for explanation), of the regression equations of the form: $\log \,k_{\text{w}} = a^{\prime}_{1} + a^{\prime}_{2} \mu + a^{\prime}_{3} \delta_{\text{Min}} + a^{\prime}_{4} A_{\text{WAS}}$, provided by the QSRR for twelve tested columns

No.	Analytical column	*a*_1_^′^	*a*_2_^′^	*a*_3_^′^	*a*_4_^′^	*r*	*s*	Fisher’s *F*-test	*p*
1	VR-150	−0.606 (±0.227)	−0.213 (±0.033) (*p* = 5E−05)	3.155 (±0.544) (*p* = 0.0001)	0.012 (±0.0006) (*p* = 3.1E−10)	0.9931	0.136	264.9	2E−10
2	NU18-125/5	−1.742 (±0.317)	−0.295 (±0.047) (*p* = 6E−05)	2.898 (±0.761) (*p* = 0.0029)	0.016 (±0.0008) (*p* = 4E−10)	0.9921	0.191	228.5	3E−10
3	NU18-250/5	−2.416 (±0.498)	−0.404 (±0.0737) (*p* = 0.0002)	2.558 (±1.194) (*p* = 0.0553)	0.017 (±0.0013) (*p* = 3E−08)	0.9839	0.299	111.0	2E−08
4	NU8-250/5	−1.724 (±0.256)	−0.192 (±0.038) (*p* = 0.0003)	2.539 (±0.614) (*p* = 0.0016)	0.014 (±0.0006) (*p* = 2E−10)	0.9926	0.154	244.3	2E−10
5	NU18-125/10	−1.512 (±0.420)	−0.308 (±0.062) (*p* = 0.0004)	2.308 (±1.006) (*p* = 0.0424)	0.016 (±0.001) (*p* = 1E−08)	0.9849	0.252	118.3	1E−08
6	UN-125	0.224 (±0.265)	−0.258 (±0.039) (*p* = 4E−05)	2.722 (±0.636) (*p* = 0.0013)	0.009 (±0.0007) (*p* = 2E−08)	0.9876	0.159	144.8	4 E-09
7	IN-150	−0.086 (±0.270)	−0.286 (±0.040) (*p* = 2E−05)	2.775 (±0.648) (*p* = 0.0013)	0.011 (±0.0007) (*p* = 5E−09)	0.9901	0.162	183.1	1. E-09
8	AC-150	0.271 (±0.173)	−0.210 (±0.025) (*p* = 5E−06)	2.460 (±0.415) (*p* = 0.0001)	0.008 (±0.0004) (*p* = 8E−10)	0.9931	0.104	262.6	2E−10
9	DI-150	−0.397 (±0.313)	−0.302 (±0.046) (*p* = 4E−05)	2.465 (±0.751) (*p* = 0.0073)	0.012 (±0.0008) (*p* = 1E−08)	0.9877	0.188	146.7	4E−09
10	DI-250	−0.039 (±0.393)	−0.220 (±0.058) (*p* = 0.0030)	3.042 (±0.943) (*p* = 0.0081)	0.011 (±0.0010) (*p* = 4E−07)	0.9763	0.236	74.5	1E−07
11	CA-125	0.165 (±0.417)	−0.295 (±0.061) (*p* = 0.0006)	2.265 (±0.998) (*p* = 0.044)	0.010 (±0.001) (*p* = 1E−06)	0.9735	0.250	66.5	2. E-07
12	LI-250	0.679 (±0.174)	−0.090 (±0.026) (*p* = 0.0049)	2.342 (±0.416) (*p* = 0.0001)	0.006 (±0.0004) (*p* = 1E−08)	0.9870	0.104	138.3	5E−09

#### Column Performance for the Determination of LH and TR along with Their Impurities

The practical test of the QSRR classification system was conducted on 12 RP-LC stationary phases during the separation of the QCs samples containing LH, TR, and their impurities (100, 200, and 300 μg mL^−1^ for LH; 0.2, 1.0, and 3.0 μg mL^−1^ for its two impurities; 250, 500, and 750 μg mL^−1^ for TR; and 0.5; 2.5, and 7.5 μg mL^−1^ for its three impurities) in accordance with the developed method described above. Next, the *t*_R_ and *R*_s_ of the compounds of interest were calculated for all RP-LC stationary phases. Furthermore, the SST values for LH and TR (the *R*_s_ minimum 5.0 between the peaks for the DMA and COX impurities, and the *R*_s_ minimum 3.0 between the peaks for TRα and TRβ, respectively) were calculated for all columns examined. These data are reported in Table 5.

**Table 5:** Summary of the retention parameters of *t*_R_ and *R*_s_ for LH, TR, and their impurities in pharmaceutical preparations, obtained under the LC-UV method for 12 tested columns

	Substances	DMA	LH		CAX		BA		DBE		TRβ		TRα		TRA		*SST*_LH_	*SST*_TR_
No.	Analytical column	*t*_R_	*t*_R_	*R*_s_	*t*_R_	*R*_s_	*t*_R_	*R*_s_	*t*_R_	*R*_s_	*t*_R_	*R*_s_	*t*_R_	*R*_s_	*t*_R_	*R*_s_		
1	VR-150	6.33	11.77	5.62	14.76	1.98	14.17	4.22	18.83	9.86	19.75	4.38	20.27	3.05	21.05	4.97	10.74	3.05
2	NU18-125/5	4.00	6.95	2.06	12.53	4.27	11.40	2.51	17.45	18.01	18.56	6.67	19.20	5.25	20.05	4.61	20.18	5.25
3	NU18-250/5	5.70	8.27	3.32	14.70	2.58	14.17	4.34	19.65	4.78	10.48	4.83	21.15	4.42	21.90	6.81	8.00	4.42
**4**	**NU8-250/5**	12.64	13.1	3.37	14.96	4.93	14.13	**0.00**	18.95	17.61	19.78	6.9	20.17	3.57	20.85	5.50	**3.63**	3.57
5	NU18-125/10	4.61	7.33	3.05	13.03	2.51	11.96	5.86	15.88	20.26	19.51	6.53	20.30	3.71	21.26	4.77	14.00	3.71
6	UN-125	5.68	9.10	4.84	13.90	2.95	13.07	7.04	19.03	26.46	20.03	9.02	20.75	6.96	21.67	8.95	17.08	6.96
**7**	**IN-150**	5.91	4.70	**0.00**	14.93	1.56	14.41	24.49	20.10	16.18	21.05	2.29	21.78	1.70	22.61	2.13	26.51	**1.70**
8	AC-150	6.65	10.67	4.73	14.35	2.62	13.73	5.61	19.58	19.94	20.80	5.64	21.50	4.40	22.28	4.95	10.61	4.40
**9**	**DI-150**	7.95	10.91	2.46	14.70	**1.37**	14.18	6.93	20.15	17.03	21.30	4.38	22.03	3.22	22.90	5.31	8.16	3.22
**10**	**DI-250**	12.48	13.10	**1.18**	16.90	1.74	16.55	16.85	22.60	33.25	23.45	6.78	24.27	6.96	24.60	2.06	16.56	6.96
11	CA-125	7.55	10.78	4.46	14.35	2.42	13.87	6.32	19.86	21.14	18.21	5.21	18.67	4.91	22.55	6.04	16.03	4.91
**12**	**LI-250**	**0.00**	**0.00**	**0.00**	15.61	1.65	15.30	**0.00**	20.90	19.50	21.70	3.43	22.47	6.04	23.30	6.39	**0.00**	6.04

Meaning of symbols is explained in the text. The columns non-suitable for the LC determination of the analytes are indicated in bold

#### Data Analysis

**Fig. 2:**
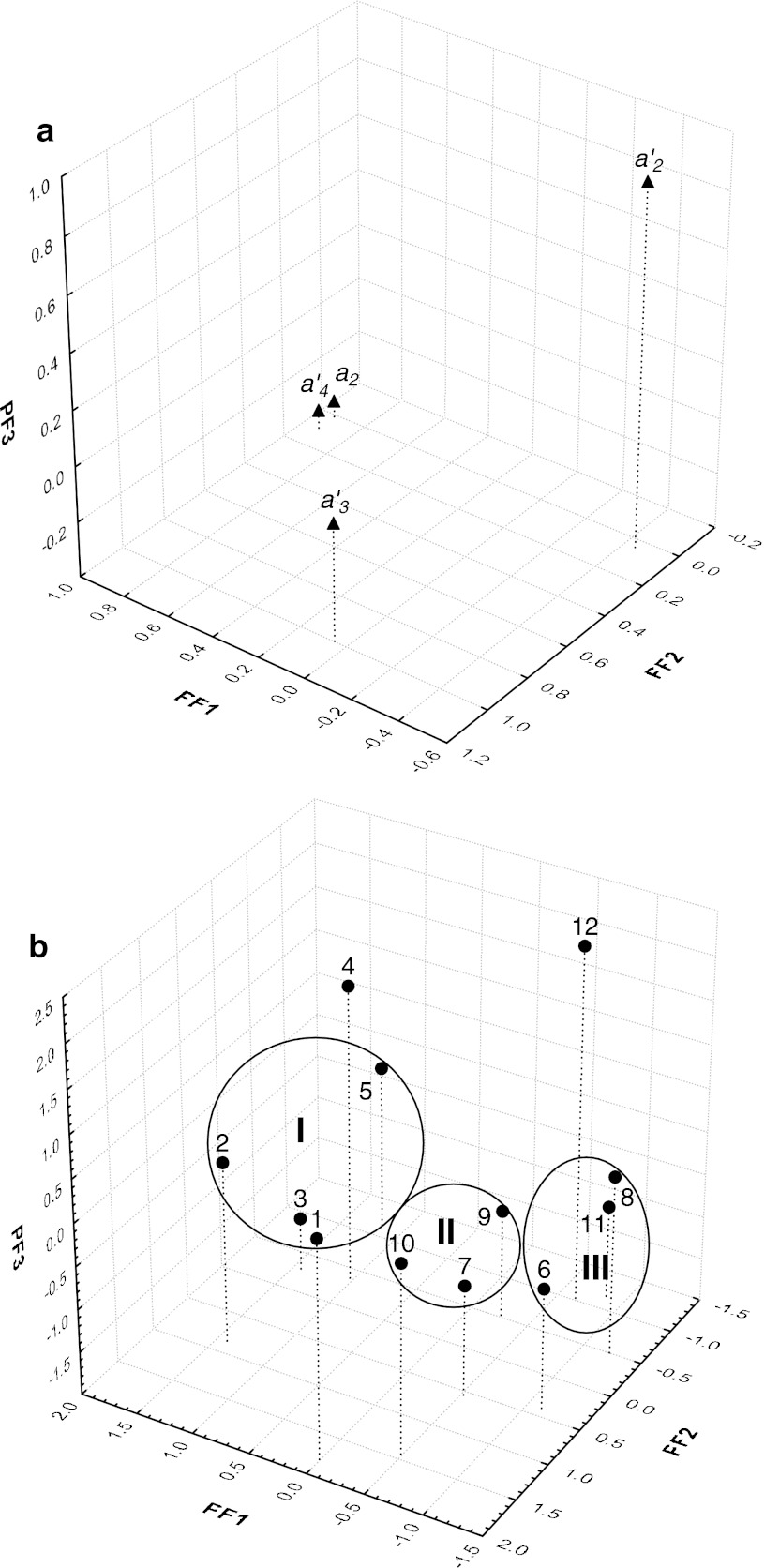
A three-dimensional FA plot for variables (**a**) and objects (**b**) based on the regression coefficients of *a*_*2*_, *a*_2_^′^, *a*_3_^′^, and *a*_4_^′^ determined in two QSRR models for 12 RP-LC columns tested

**Fig. 3:**
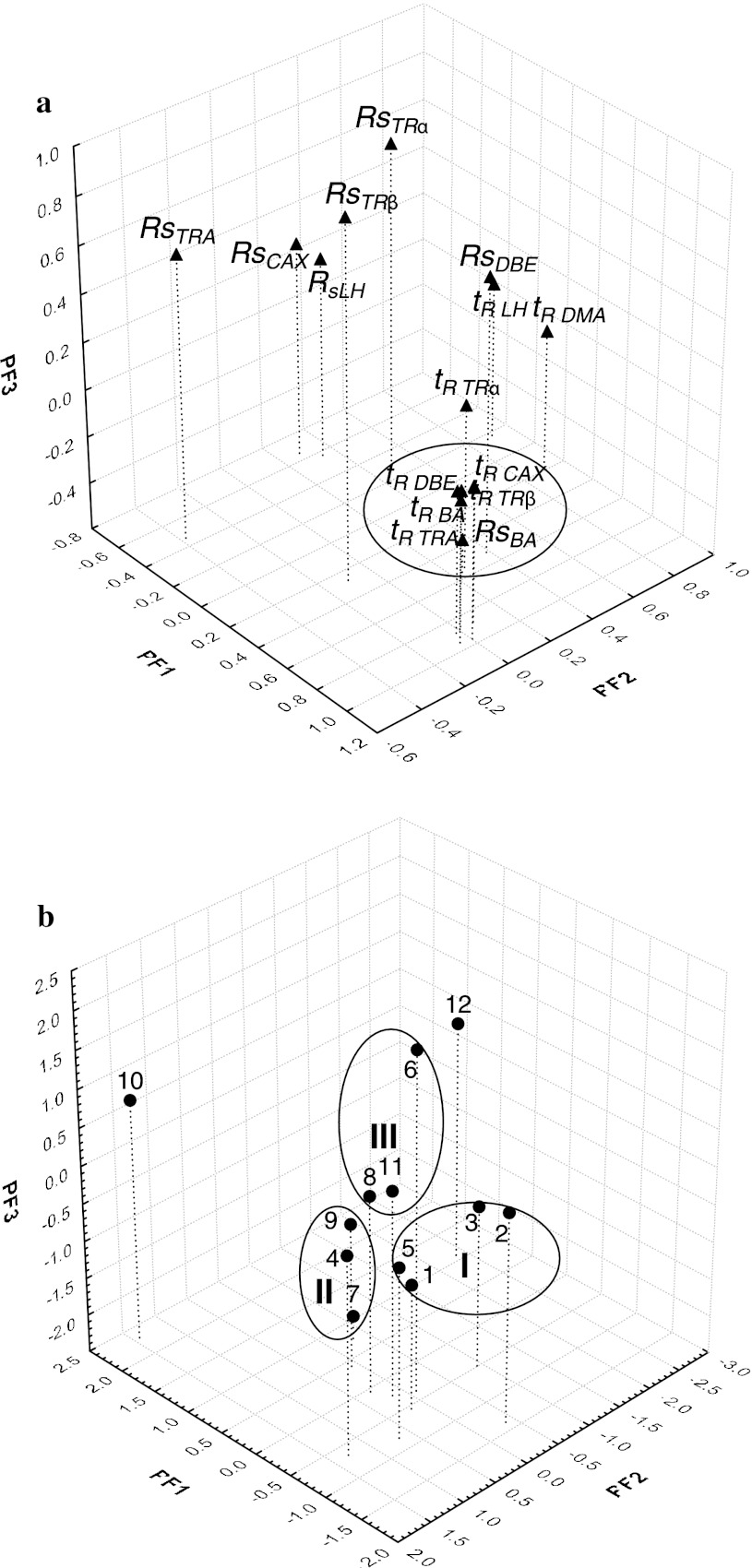
A three-dimensional FA plot for variables (**a**) and objects (**b**) based on the retention parameters (*t*_*R*_ ad *R*_*s*_) of the analytes during the column performance test for the determination of LH and TR in the presence of their impurities

The theoretical QSRR results for the 12 stationary phases and their application in pharmaceutical practice for separation of LH and TR along with their impurities were subject to a comparative study to check whether the QSRR approach could be considered a useful tool facilitating the selection of the RP-LC column. An assay employing factor analysis (FA) based on the varimax criterion was conducted to visualise the relationships between the theoretical and practical data sets. The analysis was performed on the Statistica 9.0 software (StatSoft, Tulsa, USA). The FA was first conducted based on the regression coefficients of the QSRR models: *a*_2_ from Eq.  1(Table 3) and *a*_2_^′^, *a*_3_^′^, and *a*_4_^′^ from Eq. 2 (Table 4) established for all columns. The obtained FA plots picturing the variables and the objects (columns) in three-dimensional space are shown on Fig. 2a, b, respectively. Then, the retention parameters of the *t*_R_ and *R*_s_ for LH, TR and their impurities (Table 5), calculated for all stationary phases during the column performance test were evaluated by the FA. The three-dimensional plots for the variables and columns resulting from the FA are shown in Fig. 3a, b, respectively. In the calculation, the numbers assigned to the tested columns were the same as those reported in Tables 3, 4, 5.

## Results and Discussion

As mentioned earlier on, a routine control of the pharmaceutical products containing both LH and TR requires two different analytical methods. This is both labour intensive, and cost and time consuming.

Therefore, the aim of this work was to develop a sensitive LC method for simultaneous determination of LH and TR along with their related compounds in pharmaceutical products that would be appropriate for pharmaceutical studies and drug quality control. Therefore, a series of experiments was performed to develop a simple and effective sample preparation procedure and determine the most optimal conditions for the LC analysis of the analytes.

### Optimization of Sample Preparation

The tests held to develop the best sample preparation procedure covered liquid–liquid extraction (LLE) using different organic solvents such as ethyl acetate, ether diethyl, methanol, ethanol, and mixtures of both alcohols in different proportions (from 10 to 90 % of ethanol). The assay was based on an analysis of the QC samples spiked with all compounds of interest. The obtained mean recovery data (not presented) confirmed that the LLE procedure using pure ethanol enabled the attainment of the highest efficiency and the best elimination of the ballast substances from the sample. Thus, the LLE procedure was selected for further investigations.

### Optimization of LC Separation

In the development of the presented LC method, several factors influencing chromatographic separation, such as the composition and flow rate of the mobile phase, the UV detection wavelength, and the injection volume, were tested. The LC analysis was carried out using a VR-150 column, which offers separation of all analytes at the *R*_s_ > 1.50. The best separation results were achieved with the gradient elution of 0.1 % orthophosphoric acid (solvent A) and acetronitrile (solvent B). The flow rate of 1 mL min^−1^ and the column temperature of 40 °C offering compromise between resolution and run time were found optimal for the LC separation of the analytes. It was also found that LH and its two impurities exhibited maximum absorption at 230 nm, while TR and its related compounds absorbed UV perfectly at a longer wavelength (254 nm). Therefore, different UV detection parameters were chosen as optimal for the quantification of LH and its two related compounds (230 nm) versus TR and its impurities (254 nm). Moreover, the injection volume of 30 μL was selected for further investigations because it offered low limits of detection (LOD) and good resolution of the peaks of interest.

### Validation of the Method

The chromatographic method was validated for selectivity, linearity, precision and accuracy, and recovery. The selectivity of the method was tested by analysing LH and TR in laboratory prepared mixtures in the presence of their impurities. Fig. 1a, b, respectively, show the representative chromatograms of the laboratory prepared mixtures at the concentrations of 200 μg mL^−1^ for LH and its related compounds at the level of 1 μg mL^−1^ each, 500 μg mL^−1^ for TR and its impurities at the concentration of 2.5 μg mL^−1^ each, monitored at 230 and 254 nm, respectively. Figure 1c, d, respectively, shows the representative chromatograms of the Procto-Glyvenol^®^cream containing LH (200 μg mL^−1^) and TR (500 μg mL^−1^), monitored at 230 and 254 nm, respectively. No interferences were observed between the placebo component peaks and the peaks of LH, TR, and their impurities. This proved that the proposed method was selective. The calibration curves were constructed by plotting the peak area of each analyte against the known analyte concentrations, and by performing the linear regression analysis according to the formula: y = a + bx, where y is the analyte peak area and x represents the concentration of the spiked analytes. The calibration curve equations established for all analyzed substances are summarized in Table 2. The data confirmed excellent linearity of the method for all analytes in the considered concentration range of 100–300 μg mL^−1^ for LH, 0.2–6 μg mL^−1^ for its two impurities; 250–750 μg mL^−1^ for TR, and 0.5–15 μg mL^−1^ for its three related compounds (*r* > 0.9997). The LOD and LOQ were calculated from the calibration curves using the slope (b) of the calibration graph and the standard deviation (*S*α) of the intercept (α). The intra- and inter-day precision and accuracy were assessed by analysing the QC samples tested on the same day (*n* = 6) and on six different days. Precision was expressed as relative standard deviation (RSD  %), while accuracy was assessed by calculating the estimated concentrations as the percent of the nominal concentrations. These validation results are shown in Table 2. Robustness, on the other hand, was determined by analysing the same samples under various conditions and method parameters. The impact of the mobile phase on robustness was evaluated by varying the initial percentage of the organic strength of the gradient elution between −2 and 2 %; the flow rate of the mobile phase varied in the range of 0.8–1.2 mL min^−1^, and the column temperature was changed from 33 to 43 °C. The obtained results confirmed no significant impact on the chromatographic resolutions, i.e. proved the LC method to be robust.

The proposed method was applied for the determination of LH, TR and their related compounds in two pharmaceutical preparations: Procto-Glyvenol^®^cream (Novartis, Pardubice, Czech Republic) and the Procto-Hemolan cream (Aflofarm, Ksawerów, Poland) containing 20 mg g^−1^ of LH and 50 mg g^−1^of TR, respectively. Satisfactory results were obtained for each drug (Table 2). The concentrations of all tested impurities were below the LOD.

### Column Examination

#### Column Classification by QSRR

Earlier in this report, we mentioned the well known fact that analysts often face the problem of appropriate column selection. The literature reports many column classification systems, including the QSRR, developed to solve the analytical trouble [15–28]. In the QSRR approach, each column is characterised using sets of the regression coefficient values calculated in accordance with Eqs. 1 and 2, respectively [23]. The QSRR equations obtained for the 12 stationary phases are presented in Tables 3 and 4, respectively. It should be noted that all QSRR equations, including the regression coefficients, were found statistically significant, which confirmed high quality of the QSRR results. The high correlations between log*k*_w_ and *c*log*P* were also observed what confirm similarity between the slow-equilibrium octanol/water partition system and the fast-equilibrium partition chromatographic process. Thus, hydrophobicity of the stationary phases expressed in the *a*_2_ coefficient (Table 3) was highest for NU18-250/5 and NU18-125/5, whereas its lowest values were recorded for LI-250 and AC-150, respectively.

The *a*_2_^′^ coefficients characterising the specific, polar, intermolecular interactions between the analytes and the stationary phase components exert a negative impact on the retention of the dipole–dipole (and dipole-induced dipole) attractions. The same effect is recorded in the case of the interactions between the analytes and the eluent components, hence their negative values. On the basis of the obtained *a*_2_^′^ values (Table 4), the RP-LC stationary phases can be sequenced as follows by the decreasing polarity: LI-250 > NU8-250/5 > AC-150 > VR-150 > DI-250 > UN-125 > IN-150 > NU18-125/5 > CA-125 > DI-150 > NU125/10 > NU18-250/5.

Rules similar to those described above apply to the *a*_3_^′^ coefficient, though the parameter has a positive value since it is correlated to *δ*_Min_ negative in its value (electron deficiencies). The results confirmed that the polarity of the columns examined during the study increased from CA-125 to VR-150. Coefficients *a*_3_^′^ can also be considered the parameters suitable for the evaluation of the analyte ability to take part in the hydrogen-bonding interactions with free silanols of the stationary phase support material.

When relating the *a*_4_^′^ coefficients to the *A*_WAS_ parameter one can notice their positive values for all columns. This is due to a positive impact of the non-specific analyte-stationary phase interactions on retention, and the phenomenon is due to close contact between the interacting molecules of the molecular fragments. Thus, the non-specific London retentivity caused by dispersion interactions (London interactions) puts the stationary phases in the following order according to the *a*_4_^′^ coefficient: NU18-250/5 > NU18-125/5 ≥ NU-125/10 > NU8-250/5 > VR-150 ≥ DI-150 > IN-150 ≥ DI-250 > CA-125 > UN-125 > AC-150 > LI-250.

#### Column Performance for the Determination of LH and TR along with Their Impurities

To perform the column performance test based on the measurements of LH and TR in the presence of their related compounds, the QC samples and real pharmaceutical preparations were analysed in accordance with the developed LC method involving all stationary phases. The *t*_R_ and *R*_s_ of the compounds of interest, established for all tested columns are summarised in Table 5. Notably, appropriate LC separation of all analytes according to *R*_s_ > 1.5, which is commonly accepted in LC determination, was observed for seven examined stationary phases. The LC assay of LH and the related compounds could yield proper results in accordance with the Ph. Eur. SST requirement for ten tested columns, while the SST for TR was met in the case of 11 columns. The shortest *t*_R_ of the last detected compound (TRA) versus VR-150 was obtained for NU8-250/5 and NU18-125/5, whereas LI-250 and DI-250 gave separations of the longest *t*_*R*_ values.

#### Data Analysis

The QSRR in the role of a useful tool for predicting the appropriate stationary phases for the determination of LH and TR along with their impurities was evaluated in a factor analysis (FA). The FA plots of the variables and the objects based on coefficients *a*_2_ and *a*_2_^′^, *a*_3_^′^, *a*_4_^′^ calculated for all columns (Tables 3 and 4) are illustrated in Fig. 2a, b, respectively. Here, the variance of the analysed data involved in the variability of *a*_2_ and *a*_4_^′^ (located in the same cluster—Fig. 2a) was accounted for mainly by the first primary factor (PF1: 65.11 %). The second PF2 (25.39 %) was mainly related to the variability of *a*_3_^′^, and the third one (PF3: 8.70 %) accounted primarily for the variance of the *a*_2_^′^ coefficient. These variables were found to be outliers in the plot. In the FA plot (Fig. 2b), the numbering of the columns was the same as in Tables 3 and 4. Clearly, the stationary phases positioned themselves within three subclusters grouping columns 1, 2, 3, 5 (cluster I); 7, 9, 10 (cluster II); and 6, 8, 11 (cluster III). In terms of the QSRR classification, high *a*_2_ and *a*_4_^′^ values were observed for the columns found in cluster I. In contrast, low values of both *a*_2_ and *a*_4_^′^ coefficients were characteristic for the columns located in cluster III. In the case of column Nos. 4 and 12, both found to be outliers, the QSRR yielded low and significantly low values of *a*_2_.

Figure 3b, which illustrates the FA results for the column performance test during the LC determination of LH, TR, and their impurities (Table 5), further reveals three subclusters grouping column Nos. 1–3 and 5 (cluster I); 4, 7, and 9 (cluster II), and 6, 8, and 11 (cluster III). In this plot, more than 75.23 % of the data variability was explained by the first three PFs. The location of the tested objects (columns) on the PF1 axis (41.67 %) was determined mainly by the differences in the *t*_R_ of the three TR impurities, the *t*_R_ of TRβ and the *t*_R_ of CAX. These variables were found in the same cluster (Fig. 3a). When the PF2 axis was taken into account, the positions of the columns turned out to be related primarily to the variability of the *t*_R_ of LH and DMA (19.61 %). These variables were placed close to each other, whereas the *R*_s_ of TRα and TRβ were found to be outliers on the FA plot. The variability of these variables was mainly accounted for by the PF3 (13.95 %).

The columns forming cluster I (Fig. 3b) were suitable for the LC determination of LH and TR along with their impurities. Contrary to them, the stationary phases included in cluster II gave separations with insufficient *R*_s_ for LH (No. 7), CAX (No. 9), and BA (No. 4). The stationary phases included in cluster III offered appropriate separation of the analytes at the medium values of the *t*_R_ and *R*_s_ of the compounds of interest for column Nos. 8 and 11, however lower *t*_R_ and higher *R*_s_ analyte values for column No. 6. Two stationary phases, Nos. 10 and 12, were found to be outliers on the opposite sides of the graph. Both columns were non-suitable for the LC separation of the analytes because of the insufficient *R*_s_ of LH (No. 10), no detection for the DMA and LH peaks, and the *R*_s_ of BA <1.5 for column LI-250.

Thus, the QSRR classification can be considered a useful tool in predicting realistic chromatographic separation of the analysed substances considering that the same columns grouped in clusters I and III were observed in Fig. 2b, and Fig. 3b, respectively. Moreover, in both cases, column Nos. 7 and 9 were placed in cluster II, while LI-250 (No. 12) was found to be an outlier. The only columns located in different positions on the two FA plots were Nos. 4 and 10.

## Conclusions

To summarise, a simple, precise, and accurate LC-UV method was developed for simultaneous determination of LH, TR, and their impurities in pharmaceutical preparations allowing to reduce imprecision related to sample handling, shortening the time of the analysis, and reducing its costs. Moreover, the column classification system based on two QSRR models was checked against separation selectivity of LH, TR and their related compounds. The obtained results confirmed that the QSRR approach allows for proper classification of the RP-LC columns at a relatively good level of certainty. Moreover, the study resulted in producing a list of the stationary phases for quantification of LH, TR and their impurities, which can be of interest to the pharmaceutical industry.
